# Severity of SARS-CoV-2 infection is associated with high numbers of alveolar mast cells and their degranulation

**DOI:** 10.3389/fimmu.2022.968981

**Published:** 2022-09-26

**Authors:** Olga Krysko, Joshua H. Bourne, Elena Kondakova, Elena A. Galova, Katharine Whitworth, Maddy L. Newby, Claus Bachert, Harriet Hill, Max Crispin, Zania Stamataki, Adam F. Cunningham, Matthew Pugh, Abdullah O. Khan, Julie Rayes, Maria Vedunova, Dmitri V. Krysko, Alexander Brill

**Affiliations:** ^1^ Upper Airways Research Laboratory, Department of Head and Skin, Ghent University, Ghent, Belgium; ^2^ Institute of Cardiovascular Sciences, College of Medical and Dental Sciences, University of Birmingham, Birmingham, United Kingdom; ^3^ Institute of Biology and Biomedicine, Department of Basic and Medical Genetics, National Research Lobachevsky State University of Nizhniy Novgorod, Nizhniy Novgorod, Russia; ^4^ University Clinic of Privolzhsky Research Medical University, Nizhny Novgorod, Russia; ^5^ School of Biological Sciences, University of Southampton, Southampton, United Kingdom; ^6^ Institute of Immunology and Immunotherapy, University of Birmingham, Birmingham, United Kingdom; ^7^ Department of Pathophysiology, Sechenov First Moscow State Medical University (Sechenov University), Moscow, Russia; ^8^ Cell Death Investigation and Therapy Laboratory, Department of Human Structure and Repair, Ghent University and Cancer Research Institute Ghent (CRIG), Ghent, Belgium

**Keywords:** COVID-19, mast cells, protease, LUVA cells, von Willebrand factor

## Abstract

**Background:**

The systemic inflammatory response post-SARS-CoV-2 infection increases pro-inflammatory cytokine production, multi-organ damage, and mortality rates. Mast cells (MC) modulate thrombo-inflammatory disease progression (*e.g.*, deep vein thrombosis) and the inflammatory response post-infection.

**Objective:**

To enhance our understanding of the contribution of MC and their proteases in SARS-CoV-2 infection and the pathogenesis of the disease, which might help to identify novel therapeutic targets.

**Methods:**

MC proteases chymase (CMA1), carboxypeptidase A3 (CPA3), and tryptase beta 2 (TPSB2), as well as cytokine levels, were measured in the serum of 60 patients with SARS-CoV-2 infection (30 moderate and 30 severe; severity of the disease assessed by chest CT) and 17 healthy controls by ELISA. MC number and degranulation were quantified by immunofluorescent staining for tryptase in lung autopsies of patients deceased from either SARS-CoV-2 infection or unrelated reasons (control). Immortalized human FcεR1^+^c-Kit^+^ LUVA MC were infected with SARS-CoV-2, or treated with its viral proteins, to assess direct MC activation by flow cytometry.

**Results:**

The levels of all three proteases were increased in the serum of patients with COVID-19, and strongly correlated with clinical severity. The density of degranulated MC in COVID-19 lung autopsies was increased compared to control lungs. The total number of released granules and the number of granules per each MC were elevated and positively correlated with von Willebrand factor levels in the lung. SARS-CoV-2 or its viral proteins spike and nucleocapsid did not induce activation or degranulation of LUVA MC *in vitro*.

**Conclusion:**

In this study, we demonstrate that SARS-CoV-2 is strongly associated with activation of MC, which likely occurs indirectly, driven by the inflammatory response. The results suggest that plasma MC protease levels could predict the disease course, and that severe COVID-19 patients might benefit from including MC-stabilizing drugs in the treatment scheme.

## Introduction

As of January 2022, the coronavirus disease (COVID-19) has resulted in over 343 million confirmed cases and 5,6 million deaths. The morbidity and mortality in COVID-19 patients is a result of a high virus transmission rate, inadequate anti-viral immune response, and a high incidence of life-threatening complications, such as acute respiratory distress syndrome (ARDS), extrapulmonary organ dysfunction and uncontrolled thrombosis (1–7). Understanding the mechanisms driving the development of tissue damage, ARDS and multiple organ failure in patients with severe COVID-19 is critical to develop targeted therapeutic approaches.

Mast cells (MC) are bone marrow-derived myeloid cells, which possess pleiotropic functions in allergy, asthma, anaphylactic reactions, and gastrointestinal disorders (8, 9). In healthy lung tissue, they express low levels of angiotensin converting enzyme (ACE)-2 and transmembrane serine protease 2 (TMPRSS2), two molecules involved in cellular entry of SARS-CoV-2 (10). Thus, it could be speculated that MC contribute to the severe inflammatory response and subsequent thrombosis in patients with COVID-19.

Recent studies provide increasing evidence for a role of MC in host defense. They are widely distributed in peripheral tissues and are activated in an antigen-dependent manner *via* crosslinking FcεRI in IgE-mediated allergic reactions (11, 12). In addition, MC recognize and become activated by viral and bacterial pathogens through pattern-recognition receptors (PRRs) including toll like receptors (TLRs), nucleotide oligomerization domain-like binding receptors (NLR), and retinoic acid-inducible gene-I (RIG-I)- like receptors (RIG), Mas-related G protein-coupled receptors and C-type lectin binding receptors (13, 14). Their activation induces rapid degranulation leading to the release of inflammatory mediators such as histamine, proteases, TNF-α, serotonin, heparin, bioactive lipids, cytokines and chemokines (15, 16). MC-derived mediators cause increased vascular permeability, edema and innate and adaptive immune cell recruitment (17, 18). The effects of MC on endothelial cell activation and vascular integrity could be potentiated by MC release of TNF-α and proteases. This provides a link between inflammation and blood coagulation, complement pathway, and kallikrein-kinin system (19). The mediators released during MC degranulation, including proteases and histamine, could contribute to inflammatory cell infiltration in the airways, increase of vascular permeability, and activation of airway epithelial cells (20).

MC-derived pro-inflammatory substances, such as histamine and TNF-α, can activate endothelium and stimulate its degranulation. Von Willebrand factor (VWF) is one of the mediators stored in Weibel-Palade bodies of the endothelium. This is a large protein capable of binding platelet surface glycoprotein Ibα (GPIbα) and strongly potentiating platelet adhesion and aggregation. VWF is implicated in thromboembolic events in the setting of COVID-19 (21). In the current study, we aimed to explore MC and their biomarkers in SARS-CoV-2 infection. In particular, we demonstrate increased numbers and degree of degranulation of lung MC in patients, and positive correlation of MC degranulation with lung VWF, which reflects local prothrombotic potential. We have also revealed elevated levels of MC-derived proteases in the serum of these patients, which correlates with disease severity. Finally, we report that MC are not directly activated by the whole virus or its membrane proteins. Thus, prevention of MC recruitment and degranulation may dampen the inflammatory environment and be therefore a promising therapeutic strategy in COVID-19 infection.

## Methods

### Patients

This study complies with the principles of the Declaration of Helsinki. The patients for the measurement of serum MC markers were enrolled at the University Clinic of Privolzhsky Research Medical University, Nizhny Novgorod, Russia; an approval for this research project was obtained from the local ethics committee of the Nizhny Novgorod State University. SARS-CoV-2 infection was confirmed in pharyngeal swabs by real-time reverse-transcription polymerase chain reaction (RT-PCR). The patients in the control group were age-matched healthy volunteers without any acute respiratory symptoms. A detailed description of the study cohort is provided in our earlier report (22). The patients were grouped into moderate and severe illness according to the COVID-19 treatment guidelines (23). Patients with moderate disease severity (7 males and 23 females) were admitted to hospital, but not the intensive care unit (ICU), whereas severe COVID-19 cases (11 male and 19 female) were both admitted to hospital and ICU. Postmortem MC staining in the lungs was performed at the University of Birmingham. Collection of post-mortem FFPE tissue was approved (IRAS: 197937) and the samples were received by a prospective consent and retrospective acquisition of tissue, for which consent for use in research had already been obtained. Ethical approval for the use of patient tissue was provided by the Health Research Authority with a National Health Service Research Ethics Committee; approval issued by North East-Newcastle and North Tyneside 1 (19/NE/0336). A written consent was obtained for all patients. Patient (n = 8) lung samples were taken from individuals deceased from COVID-19 infection, aged between 59 and 89, of both sexes. Controls (n = 3) were individuals died from non-COVID-19-related reasons. Detailed information about both patient and control cohorts can be found in (24). Patient lung damage was quantified by a semi-quantitative scale for the evaluation of lesion volume in lungs based on a chest CT scan, applied according to the national guidelines (25, 26) as follows: chest CT grade 0 (no low abnormalities on the chest CT), chest CT grade 1 (25% of the lungs were affected), chest CT grade 2 (50% of the lungs were affected), chest CT grade 3 (75% of the lungs were affected), chest CT grade 4 (100% of the lungs were affected).

### MC staining in the lungs

FFPE sections were deparaffinized and rehydrated, followed by antigen retrieval by heating in a microwave (10 min) in citrate buffer (pH 6). Autofluorescence was quenched using 3% hydrogen peroxide and sections blocked by PBS-Tween 0.05% containing 3% bovine serum albumin (BSA) and 5% goat serum for 1h at room temperature. Monoclonal primary antibody against tryptase (1:1000, clone G3, Merck) was applied overnight at 4°C. Then, the sections were washed, incubated with secondary antibody conjugated with Alexa-Fluor 647 (Invitrogen), washed again and autofluorescence was quenched again by Vector^®^ TrueVIEW^®^ Autofluorescence Quenching Kit (Vector Laboratories, CA, USA). After mounting, sections were analyzed by an epifluorescent microscope. Tryptase-positive objects with the area of >50 μm were considered MC, whereas objects with the area of <50 μm were regarded as released granules. The numbers of lung MC and cell-free granules as well as MC area were averaged from 15 random fields per patient/control using ImageJ (National Institute of Health, USA) (27).

### VWF staining in lung sections

FFPE lung sections from COVID-19 patients and controls were rehydrated and underwent antigen retrieval and blocking with PBS containing 5% BSA and 10% goat serum for 1h at room temperature. Next, sections were incubated overnight with rabbit anti human VWF antibody (Agilent) at 4°C and then secondary antibody (goat anti rabbit, conjugated to Alexa Fluor 647, Invitrogen, 1 h, RT). Nuclei were counterstained by DAPI. Lung autofluorescence was quenched using commercial kit (Vector laboratories) and slides mounted using ProLong Gold Antifade Mountant (Life Technologies). VWF analysis was performed as described (28). Briefly, images were acquired using a Zeiss Axio Scan.Z1 Epi Fluorescence system and quantified using Fiji (29) by manually designating ROIs and measuring intensity after background subtraction and denoising.

### Enzyme-linked immunosorbent assays

On the day of admission to the hospital, peripheral blood samples were collected by venipuncture, centrifuged for 10 min at 845 g, and sera were stored at −80°C until further analysis. The levels of MC proteases in sera of the patients were analyzed by commercial ELISAs for human tryptase beta-2 (TPSB2, ELH-TPSB2-1, RayBio, Peachtree Corners, GA, USA), chymase 1 (CMA1, ELK3058, ELK Biotech, Wuhan, P.R.C), and carboxypeptidase 3 (CPA3, LS -F52226, LSBio, Seattle, WA, USA).

### Biochemical parameters

The analysis of creatinine, glucose, alanine aminotransferase (ALT), aspartate aminotransferase (AST), C-reactive protein (CRP), were done by an Indiko automatic biochemical analyzer (ТhermoScientific, Finland) using the manufacturer’s reagents. Control materials were produced by RANDOX (Randox Laboratories, UK). Lactate dehydrogenase activity in serum was analyzed using a kinetics method according to the manufacturer’s instructions (DDS *in vitro* Solutions, Pushchino, Russia). The levels of fibrinogen and D-dimer were analyzed using a coagulation analyzer ACL TOP 500 (Instrumentation Laboratory, USA).

### Cell culture

Immortalized human mast cell line, LUVA (Kerafast, USA), was used to model lung-resident mast cell response to SARS-CoV-2 infection. We and others (Laidlaw et al., 2011) have shown that LUVA cells express FcεRI and c-Kit similarly to normal human MC. Cells were cultured in StemPro-34 SFM media (Gibco, USA) with L-glutamine (2 mM) and Penicillin-Streptomycin (10 U/ml) in a humidified incubator at 5% CO_2_ and 37°C. Cells were maintained at 5x10^5^/ml and passaged up to 15 times; FcεRI and c-Kit expression the cell surface of LUVA cells was maintained throughout passaging.

### Antigen generation and virus

SARS-CoV-2 England 2 virus (Wuhan strain) was a kind gift from Christine Bruce, Public Health England. Recombinant trimeric spike glycoprotein (S) was produced in human embryonic kidney (HEK) 293F cells as previously described (30). Nucleocapsid (N) protein was produced in *E.coli* bacteria and purified as described (31). The levels of endotoxin in the protein preparations were lower than 0.005 EU/mg.

### MC stimulation

LUVA cells were either naive or primed using the presence or absence of native human IgE (1 µg/ml; Abcam, UK) for 24 h, before thrice washing with HEPES buffer. Cells were plated at 3x10^5^/ml, and stimulated with a vehicle control, Compound 48/80 (50 µg/ml; Sigma) or SARS-CoV-2-related antigens (1-10 µM) for 1 h at 37°C in a CO_2_-free incubator; CO_2_ has been shown to limit MC degranulation (32). The stimulation of LUVA cells by SARS-CoV-2 (2,500 IU/ml) were performed as above, but at 5% CO_2_ due to limitations by Biosafety level 3.

### Flow cytometry

LUVA cells were blocked in 10% serum (20 min on ice), prior to an incubation in the conjugated antibody cocktail, or company recommended isotype control (30 min on ice): hAce-2-AF405 (#535919; 1:300; R&D, USA), Avidin-fluorescein (#A821; 1:5,000; Invitrogen, USA), FcεRIα-PE (#AER-37; 1:300; Invitrogen, USA), CD63C-PECy5.5 (#H5C6; 1:300, BioLegend, USA), CD203c-PECy7 (#NP4D6, 1:300, Invitrogen), c-Kit-APC (#104D2; 1:300; BD Bioscience, USA). Cells were fixed with 10% formalin, and acquired using a CytoFLEX flow cytometer (Beckman, USA). At least 10,000 events were recorded, and gating strategy is outlined in **Figure S1**. Data was analyzed using FlowJo v10 and presented using GraphPad Prism v9.

### Statistical analysis

The difference between the healthy controls, moderate and severe patients was analyzed by one-way ANOVA and Kruskal-Wallis test for multiple comparisons. Following analysis for Gaussian distribution, Pearson’s correlation coefficient and two-tailed p values were calculated for the selected datasets using GraphPad Prism 9.0. The numbers of lung MC, cell-free granules, and MC area were compared by two-tail student’s t-test. The p-value of less than 0.05 was considered significant.

## Results

### Clinical characteristics of the COVID-19 patient cohort

Patients were classified based on the severity of SARS-CoV-2-infection as described in the Methods. Patients with severe disease were significantly older than moderate cases (59.1 ±15.9 *vs*. 47.7 ±16.2 years, p < 0.02) with mildly increased body mass index (31.6 ±4.9 *vs.* 27.8 ±4.7, p=0.003) (**Table 1**). No marked difference in the symptoms was seen on admission between patient groups while severely affected patients had several comorbidities as described in our earlier report (22). On admission, severely ill patients with COVID-19 had a higher breathing rate than moderately ill patients. Peripheral capillary oxygen saturation (SpO_2_) reached 95.2 ±1.9% in moderate cases and was lower in severe cases (89.9 ±5.3%, p<0.0001). Fifteen patients with severe COVID-19 received non-invasive oxygen support, while those with a moderate severity did not require oxygen support.

**Table 1:** Demographics and baseline characteristics of patients included in the study.

	controls	moderate	severe
	(n=17 )	(n=30)	(n=30)
Age, years	54,7±19,7	47,7±16,2	59,1±15,9
Gender	7M/10F	7M/23F	11M/19F
Body mass index	25,09±3,6	27,8±4,7	31,6±4,9
Hypertension (n,%)	4 (23%)	16 (53%)	24 (80%)
Diabetes (n,%)	1 (6%)	1 (3%)	13 (43%)
Cardiovascular disease (n,%)	3 (17%)	4 (13%)	14 (46%)
Malignancy (n,%)	0 (0)	4 (13%)	5 (16%)
Stroke (n,%)	0 (0)	1 (3%)	3 (10%)
Chronic lung diseases (n,%)	0 (0)	2 (6%)	2 (6%)
Arrhythmia (n,%)	0 (0)	4 (13%)	1 (3%)
Rheumatoid arthritis (n,%)	1 (6%)	0 (0%)	1 (3%)

The number (n) of the patients in each group is provided with description of their age, gender, and comorbidities. For demographic characteristics data are presented as median ± standard deviation.

Fifty-six percent of the patients with severe COVID-19 and only 16% of patients with moderate disease had thromboembolic events such as stroke and myocardial infarction. A chest CT scan performed upon patient admission revealed pneumonia in 27 out of 30 severely ill COVID-19 patients. Ten percent of the patients with moderate disease severity had 75% lung damage accompanied by moderate clinical symptoms, 63% had lung damage between 25 and 50%, and 27% had below 25%. Only one patient in the group with moderate COVID-19 had reported current tobacco smoking. The patients were hospitalized for a median of 9.1 ±6 days to 9 ±3.6 days for severely and moderately ill COVID-19 patients, respectively. Four patients in a group of severely affected patients were mechanically ventilated and eventually died from ARDS and endotoxic shock.

### MC proteases are elevated in patient serum relative to the clinical severity of COVID-19

**Figure 1:**
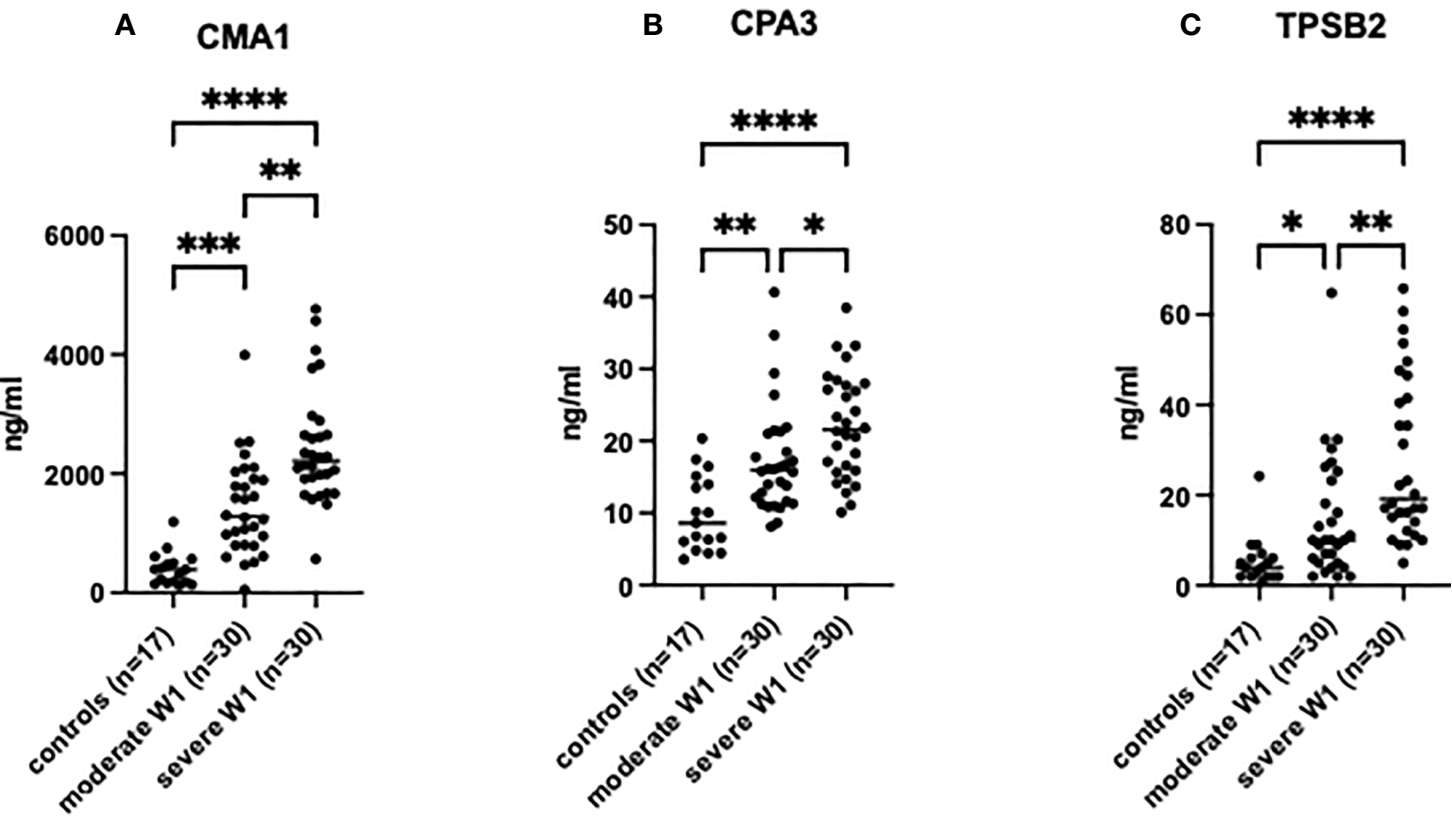
MC degranulation markers in patient serum are increased relative to clinical severity of patients post-SARS-CoV-2 infection. The presence of **(A)** CMA1, **(B)** CPA3 and **(C)** TPSB2 in patient serum was determined by ELISA. Healthy controls (n=17) were used to compare to patients post-SARS-CoV-2 infection with moderate (admission to hospital; n=30), or severe (admission to ICU; n=30) clinical outcome. Statistical significance was determined using a one-way ANOVA with Kruskal-Wallis multiple comparison test. *p<0.05, **p<0.01, ***p<0.001, ****p<0.0001.

The MC protease levels were analyzed in the serum of patients on the day of admission. All tested MC proteases, CMA1, CPA3, and TPSB2, were increased in the serum of patients compared to healthy controls (**Figure 1**), and in severe patients compared to moderate individuals. The levels of the proteases in four patients subsequently deceased from COVID-19 were comparable to the levels in the patients with severe COVID-19: CPA3 (21.54 ±6.6 *vs*. 22.25 ±5.7 ng/ml, p = 0.72); CMA1 (2426 ±1113 *vs*. 2387 ±720 ng/ml, p = 0.73) and for TPSB2 (27.5 ±17 *vs*. 30.04 ±17 ng/ml, p=0.95). Fifteen patients with severe COVID-19 that required oxygen support had levels of MC proteases comparable to other patients with severe disease (**Figure S1**).

A strong positive correlation was observed between the degree of lung damage (4 grades assessed by chest CT scan) and the serum levels of CMA1 (Pearson’s r= 0.591, p<0.0001), CPA3 (Pearson’s r= 0.577, p<0.0001), and TPSB2 (Pearson’s r= 0.503, p<0.0001) (**Table 2**). Levels of MC proteases in serum of patients with COVID-19 strongly correlated with the severity grade of the disease (**Table 2**). Patients with severe COVID-19 showed higher levels of proteases than individuals with moderate severity. Thus, MC protease activity is associated with lung damage and subsequent poor clinical outcome post SARS-CoV-2 infection although certain contribution of other comorbidities in SARS-CoV-2 patients cannot be ruled out.

**Table 2:** Correlation analysis of MC protease levels in serum with demographical and clinical characteristics of patients with COVID-19.

		CMA1		CPA3		TPSB2	
		Pearson’s r	p-value	Pearson’s r	p-value	Pearson’s r	p-value
**Age, year**		0.147	0.261	0.209	0.11	0.127	0.335
**Body mass index**		0.109	0.417	0.121	0.368	0.281	0,033
**Intensive care unit**		0.187	0.153	0.244	0.06	0.3	0,020
**Oxygen support**		0.174	0.183	0.3	0,020	0.125	0.341
**Severity grade**		0.561	<0,0001	0.452	<0,0001	0.465	<0,0001
**Chest CT at admission (grade)**		0.591	<0,0001	0.577	<0,0001	0.503	<0,0001
**Chest CT at day 7-10**		0.586	<0,0001	0.561	<0,0001	0.472	<0,0001
**(grade)**							
**SpO2 at admission**		-0.076	0.564	-0.214	0.102	-0.399	0,002

Pearson correlation coefficient and two-tailed *p* values were calculated for the selected datasets using GraphPad Prism 9.0.

### MC numbers and degranulation are increased in the lungs of COVID-19 patients

**Figure 2:**
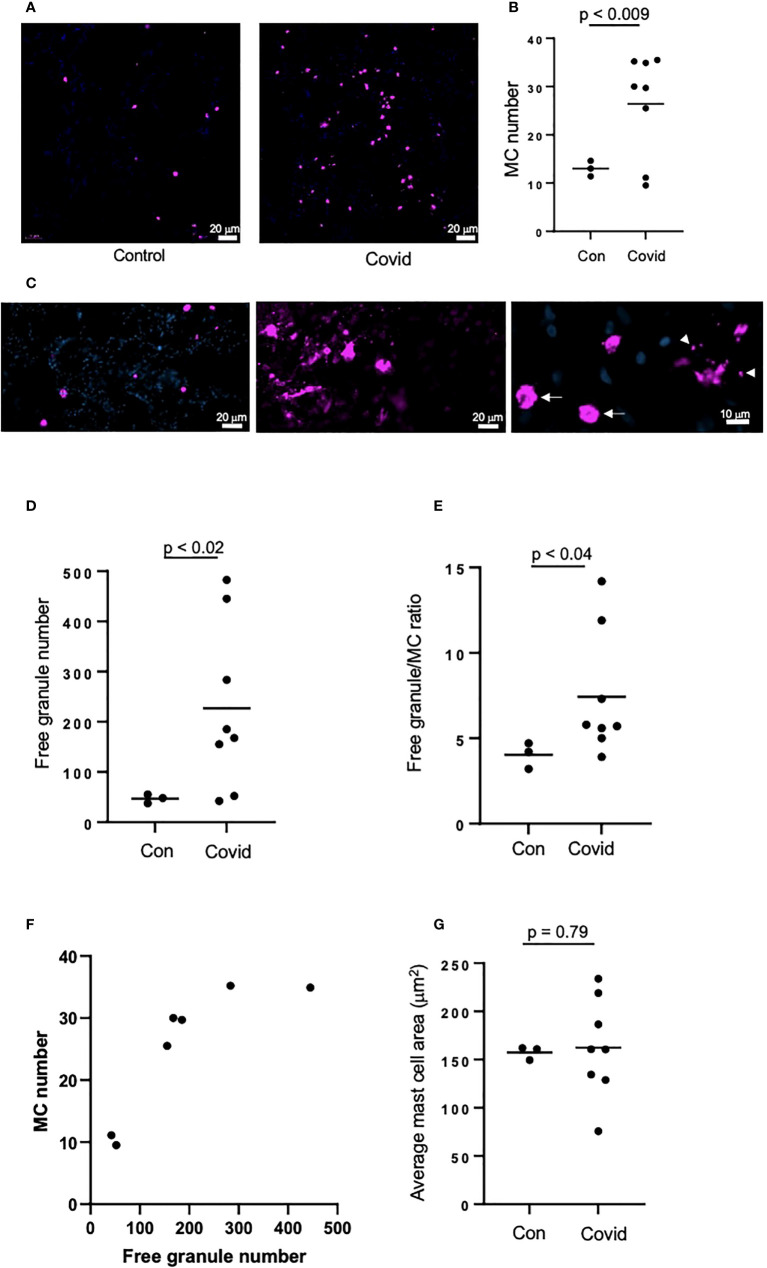
MC recruitment and degranulation is increased in the lungs of patients post-SARS-CoV-2 infection. The lungs of patients deceased post-SARS-CoV-2 infection were frozen, sectioned to 6 μm and labelled by immunofluorescence, before imaging using an epifluorescence microscope. Lungs of non-SARS-CoV-2-linked deaths were used as a control. **(A)** MCs were identified by tryptase (magenta) in the lungs of deceased non-SARS-CoV-2 (n=3) or COVID-19 patients (n=8); **(B)** Quantification of MC numbers; **(C)** Non-degranulated (controls, left panel) and degranulated (COVID-19, middle panel) MCs; right panel represents non-degranulated MC (arrows) and granules (arrowheads) releasing from a MC in the process of degranulation; **(D)** Quantification of MC granules identified as tryptase-positive objects with the area of <50 µm^2^; **(E)** Ratio between the numbers of cell-free granules and MCs; **(F)** Correlation between the numbers of MCs and cell-free granules; **(G)** Quantification of the average of MC area. Images were analyzed using ImageJ. Horizontal lines represent mean. Statistical significance was determined using the unpaired Student’s t-test.

We next assessed MC accumulation and degranulation in lung autopsies by immunofluorescence staining for MC marker, tryptase. These sections were collected from a separate cohort of patients who died of COVID-19, as well as from patients who died from non-COVID-19-related reasons as a control. The numbers of tryptase-positive MC (**Figures 2A, B**) were dramatically increased in patients with COVID-19 *vs.* non-COVID-19 controls (26.4 ±9.85 *vs.* 13 ±1.32 per view field, p < 0.009). The amount of secreted MC granules was also elevated as well as the granule/MC ratio, which reflects the average number of released granules per one MC (226.7 ±154.5 *vs.* 47.1 ±7.28 per view field, p < 0.02 and 7.3 ±3.4 *vs.* 4 ±0.63 per view field, p < 0.04, respectively; **Figures 2C–E**). There was a strong correlation between the numbers of MC and secreted granules (r = 0.84, p < 0.02; **Figure 2F**). Enhanced release of granular content could be expected to reduce the volume of MC; hence we next calculated the average area of MC in the lung sections, however this parameter did not differ between COVID-19 patients and controls (**Figure 2G**). The increased numbers of MC and their granules in the lungs post-infection may suggest their contribution to the immune response in patients with COVID-19.

### Neither live SARS-CoV-2 nor its viral proteins directly activate MC *in vitro*

**Figure 3:**
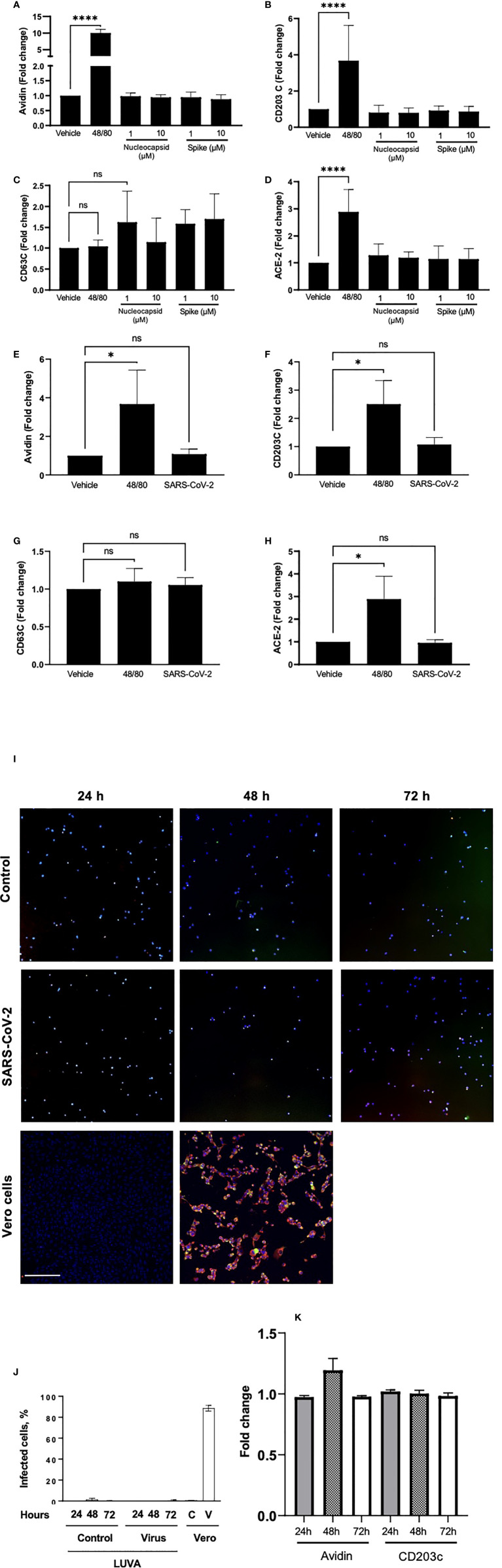
Neither viral proteins nor the SARS-CoV-2 virus directly induce MC degranulation, and SARS-CoV-2 virus does not penetrates MC. LUVA cells were cultured in the presence or absence of compound 48/80 (10 µg/ml) or spike protein or nucleocapsid protein (1-10 µM; **A–D**), or SARS-CoV-2 **(E–H)** for 1 h in a CO_2_-free humidified atmosphere at 37°C. Cells were analyzed for the expression of **(A, E)** avidin, **(B, F)** CD203c, **(C, G)** CD63, or ACE-2 **(D, H)** using a CytoFlex flow cytometer and quantified using FlowJo v10. Statistical significance was determined using a one-way ANOVA with Tukey’s multiple comparisons test. *p<0.05, ****p<0.0001. ns, not significant. LUVA cells were incubated without or with (first and second rows, respectively) ARS-CoV-2 virus for 24 h, 48 h or 72 h, after which cells were seeded on a polylysine-coated polystyrene wells and stained for nuclei (blue), nucleocapsid protein (red), and spike protein (green) to evaluate virus entry into the cells **(I)**. Vero cells (third row) served as a control of both virus penetration and quality of the antibodies. No dissemination of the virus inside cells was observed at all time points, which is confirmed by quantitation of the fluorescence **(J)**; C and V designate control and virus for Vero cells. Staining of cell samples for avidin and CD203c revealed no degranulation **(K)**. Scale bar 200 um, n = 3 for each time point.

To test whether SARS-CoV-2 can directly activate MC, we next performed experiments using human immortalized MC line, LUVA, initially derived from CD34^+^ mononuclear cells (33). Prior to the experiments, we confirmed that the cells express high levels of both CD117 (c-kit) and FcεRI, typical for a regular MC phenotype (**Figure S2**). Incubation of LUVA cells with increasing concentrations of SARS-CoV-2 viral proteins nucleocapsid or spike protein for 1 h, as well as live SARS-CoV-2, did not change the expression of both FcεRI and CD117 (data not shown). The proteins also did not affect MC degranulation as assessed by granule staining using fluorescently-labeled avidin (**Figure 3A**), which rapidly binds heparin in MC granules (34). The membrane expression of CD203c or CD63 (**Figures 3B, C** **)** as well as by β-hexosaminidase secretion were unchanged (data not shown). Stimulation of MCs with compound 48/80, a potent MC secretagogue, upregulated the surface expression of ACE-2, whereas its expression remained unchanged in the presence of the viral proteins (**Figure 3D**). Expression of CD63, CD203c, ACE-2, and avidin binding remained unaffected also after exposure of the cells to the live virus (**Figures 3E–H**). The same results were obtained also in all described experiments using MCs primed with native human IgE (data not shown). Incubation of LUVA cells with the virus for longer periods of time (24 - 72 h) also resulted in neither virus penetration into the cells, nor their activation (**Figures 3I–K**). Thus, degranulation of MCs post-SARS-CoV-2 infection is unlikely to be through a direct activation by the virus but could occur through mechanisms different from a direct contact with viral epitopes.

### MC granule release positively correlates with lung VWF levels in COVID-19 patients

**Figure 4:**
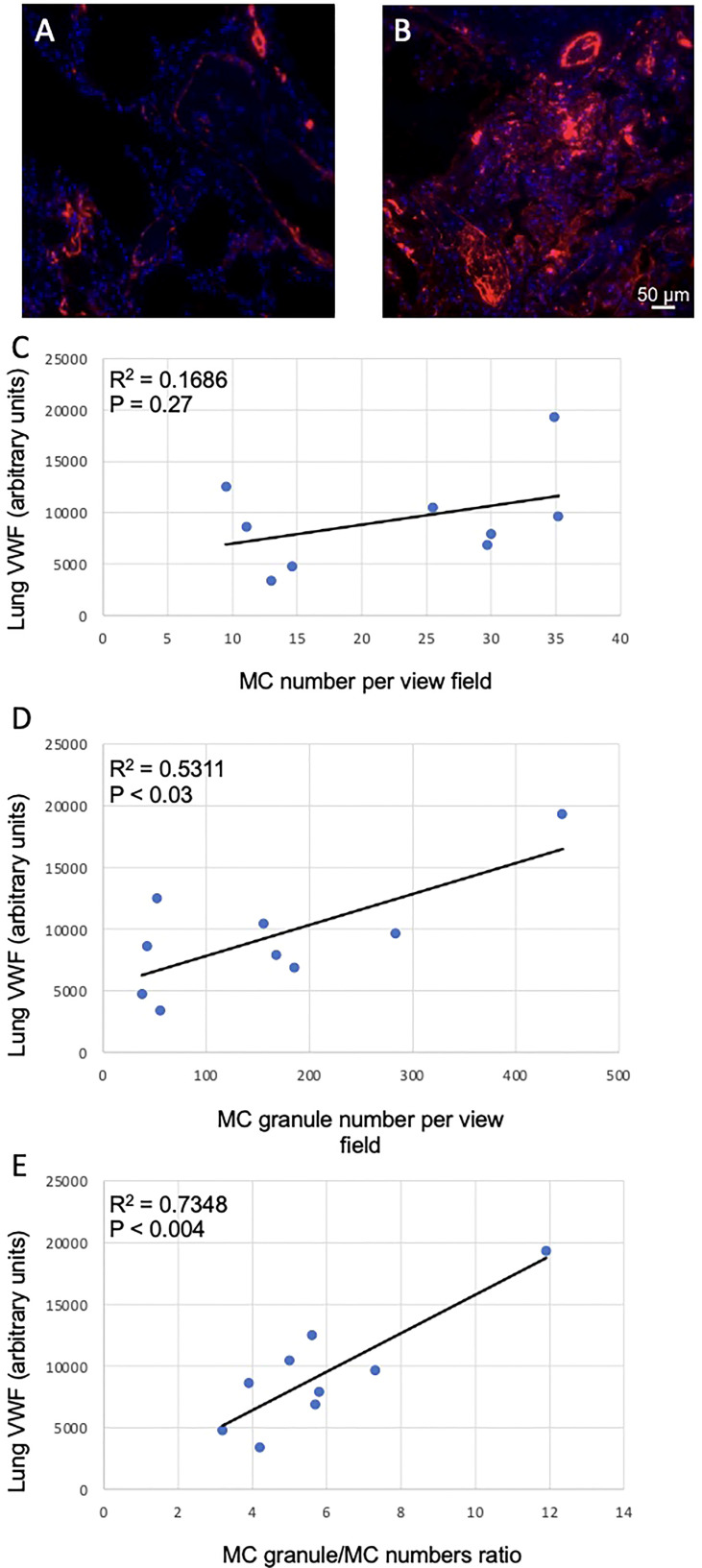
Correlation between MC-related parameters and lung VWF levels. The levels of VWF in the lungs were determined as fluorescence intensity in arbitrary units. **(A)** and **(B)**, representative images of VWF staining in the lungs of non-COVID-19 and COVID-19 patients, respectively. Correlations of VWF levels with **(C)** MC numbers, **(D)** cell-free granule numbers and **(E)** granule/MC number ratio are presented. Pearson r coefficient was calculated using MS Excel.

COVID-19 infection frequently leads to thrombotic complications. To address whether MCs could be implicated in the development of local pro-thrombotic conditions we assessed the correlation of VWF levels in the lung sections (**Figures 4A, B**) with MC-related parameters. No correlation of lung VWF with MCs numbers was observed in the lungs of patients with COVID-19 (p = 0.27; **Figure 4C**). In contrast, the number of secreted granules moderately correlated with the VWF levels (r = 0.5311, p < 0.03) whereas correlation with granule/MC ratio was strong (r = 0.7348, p < 0.004; **Figures 4D, E** **)**. Thus, the degree of MC degranulation rather than their total numbers is associated with the pro-thrombotic phenotype typical of SARS-CoV-2 infection.

## Discussion

In the current study, we have analyzed the role of MC activation post-SARS-CoV-2 infection by measuring the levels of three MC-specific proteases, TPSB2, CMA-1, and CPA3, in serum of patients with moderate and severe clinical severity. We report (i) increased levels of MC proteases in serum of patients with COVID-19, and (ii) enhanced infiltration of MCs and their degranulation in lung tissue post-mortem, which may imply MC involvement in the pathogenesis of the disease. The degree of degranulation of each MC was increased and positively correlated with lung VWF levels; (iii) the live SARS-CoV-2 virus or its protein components did not degranulate MCs *in vitro*, suggesting that an indirect mechanism of MC activation could be involved.

Our studies show the simultaneous increase in CMA1, CPA3, and TPSB2 in COVID-19 patients, which are indicative of the MC_TC_ subset of MCs (the most common subset in human airways) in the lungs of patients with COVID-19 (35). Eosinophil and MC activation has previously been reported in patients with COVID-19, whereby MC proteases in serum and lungs of COVID-19 patients were elevated (36). TPSB2 and CMA-1 have been demonstrated to induce vascular leakage in response to viral infection, which may explain the increased lung damage observed in CT scans (37). Moreover, other mediators released during MC degranulation, including proteases and histamine, could contribute to inflammatory cell infiltration in the airways, increase of vascular permeability, and activation of airway epithelial cells (20). Moreover, increased CMA1 in patients with severe COVID-19 contributes directly to destabilization of cellular contacts *via* degradation of MMPs, laminin, fibronectin (38, 39) and to the generation of pro-inflammatory cytokines IL-6, IL-1β, IL-18, IL-33, and TNF-α (40–43). This has been supported by *in vivo* data, whereby the administration of MC stabilizers reduced SARS-CoV-2-induced production of pro-inflammatory factors and the degree of lung injury (20). This outlines a clear role for MCs and their proteases in the modulation of the inflammatory response to SARS-CoV-2 through cytokine production and regulation of vascular permeability.

We here demonstrated that MC numbers increase in the lungs of patients with COVID-19. These findings corroborate previously reported accumulation of MCs in the perivascular spaces and alveolar septa next to alveolar capillaries (44) as well as recently demonstrated MC-mediated lung tissue damage (45). In the setting of SARS-CoV-2 infection, MCs accrued in the lungs had a connective tissue phenotype, which is untypical of non-COVID-related lung infections (45). In our samples, MCs were apparently activated as large amounts of cell-free tryptase-rich granules were observed in the lung tissues. Moreover, the total amount of the released MC content was apparently defined not only by the elevated numbers of MCs *per se*, but also by increased numbers of granules released by each MC as judged by the ratio between the quantities of MCs cell-free granules.

The nature of the factors recruiting MCs into the lungs is still to be defined. The “cytokine storm” observed during SARS-CoV-2 infection includes multiple pro-inflammatory agents that can chemoattract MC, such as components of the coagulation system (*e.g.*, fibrinogen) or TGF-β, shown to contribute to pulmonary fibrosis (46–48). Multiple agents found in the plasma of COVID-19 patients, such as TNF-α and IL-8, can induce chemotaxis of MC. VWF is one of the key prothrombotic proteins capable of recruiting platelets, which in turn can stimulate MC degranulation through platelet-activating factor (49). VWF is stored in the Weibel-Palade bodies of the endothelium, and it is likely that MC-derived histamine, one of the most potent Weibel-Palade bodies secretagogues, stimulates its release in the COVID-19 setting. It has recently been shown that thrombi in the pulmonary artery can develop *in situ* (50), a process, in which VWF could be involved in the COVID-19 setting. Immuno-thrombosis mediated by MC accumulated in the lung septa has recently been reported using histopathology of post-mortem lung biopsies of patients with COVID-19 (44). This could represent a “vicious circle” underlying massive pulmonary thrombosis, a leading cause of SARS-Cov-2-induced mortality.

It is reasonable to hypothesize that MC degranulation is directly induced by viral proteins. Several viruses were shown to induce MC degranulation, including influenza A, Zika virus, herpes simplex virus and respiratory syncytial virus (51–56). Despite previous reports that the SARS-CoV-2 virus induces degranulation of MC line, LAD2, and in humanized ACE-2 mice (20), we did not observe direct LUVA MC activation by the virus or its proteins. Exposure of LUVA cells to live virus for longer periods of time (days) resulted in neither virus replication in the cells nor cell degranulation. This finding suggests that, in COVID-19 infection, MC are likely activated indirectly, potentially by certain elements of the “cytokine storm” typical for this infection, which are obviously absent in the cell culture experiments. It cannot also be ruled out that specific microenvironment in the lungs renders MC more susceptible to direct interactions with viral components than in the *in vitro* model. MC activation by a secondary mediator likely facilitates its binding to the virus, as judged by elevated expression of ACE-2 induced by compound 48/80, although the consequences of such binding are still to be determined. MC activating agents could originate from immune cells such as macrophages, which are considered critical for cytokine storm, COVID-19 severity and eventually death (57, 58). It is conceivable that MC degranulation can further accelerate lung inflammation and eventual dysfunction through release of histamine, myeloid chemoattractant CCL3 and prostaglandins (11). Therefore, MC could be a key point for the positive feedback, in which they are activated by a strong systemic pro-inflammatory milieu and then further enhance local inflammatory response.

In conclusion, our study shows that activation and degranulation of MC in the lungs of patients with COVID-19 correlates with the degree of disease severity, lung damage and local pro-thrombotic potential. An increase in MC protease levels is more pronounced in severe COVID-19, which could potentially contribute to their detrimental role in disease progression by increasing vessel permeability and contributing to the progression of ARDS and cytokine storm *via* secretion of inflammatory mediators. Therefore, the use of MC stabilizers might be beneficial in dampening the inflammatory response in patients at risk of developing severe COVID-19.

## Data availability statement

The raw data supporting the conclusions of this article will be made available by the authors, without undue reservation.

## Ethics statement

The studies involving human participants were reviewed and approved by the Health Research Authority (HRA) with an NHS (National Health Service) REC (Research Ethics Committee); approval issued by North East-Newcastle and North Tyneside 1 (19/NE/0336). The patients for the measurement of serum MC markers were enrolled at the University Clinic of Privolzhsky Research Medical University, Nizhny Novgorod, Russia; an approval for this research project was obtained from the local ethics committee of the Nizhny Novgorod State University. The patients/participants provided their written informed consent to participate in this study.

## Author contributions

OK, AB, MV, and DK designed experiments, analyzed data, supervised the study, and wrote and revised the manuscript; JB performed experiments, collected and analyzed data and revised the manuscript; KW, AB, HH, and ZS performed experiments; EG enrolled patients in the study, collected clinical information and peripheral blood; AK analyzed data; JR performed experiments, and revised the manuscript. MC, MN, MP, and AC provided reagents and revised the manuscript. EK performed ELISA’s, analyzed data and revised the manuscript; CB revised the manuscript. All authors contributed to the article and approved the submitted version.

## Funding

The research was supported by FWO-Flanders 3G065319N to OK and CB. DK lab is supported by FWO-Flanders (G043219N, G016221), Ghent University BOF (Special Research Fund 01/O3618 and BOF/IOP/2022/033) and the project (40007488) from the FWO and F.R.S.-FNRS under the “Excellence of Science” program. MV acknowledges the Ministry of Science and Higher Education of the Russian Federation, agreement No. 075-15-2020-808. JR is a British Heart Foundation Intermediate Fellow (FS/IBSRF/20/25039). AK is a Henry Wellcome fellow (218649/Z/19/Z). The study was supported by the British Heart Foundation Senior Basic Science Research Fellowship (FS/19/30/34173) and a BHF Project Grant (PG/18/46/33817) to AB.

## Conflict of interest

The authors declare that the research was conducted in the absence of any commercial or financial relationships that could be construed as a potential conflict of interest.

## Publisher’s note

All claims expressed in this article are solely those of the authors and do not necessarily represent those of their affiliated organizations, or those of the publisher, the editors and the reviewers. Any product that may be evaluated in this article, or claim that may be made by its manufacturer, is not guaranteed or endorsed by the publisher.
