# Supplementary figures and images for: Severity of SARS-CoV-2 infection is associated with high numbers of alveolar mast cells and their degranulation
Source: Front Immunol. 2022 Sep 26;13:968981. doi: 10.3389/fimmu.2022.968981 (PMC9548604; doi:10.3389/fimmu.2022.968981)

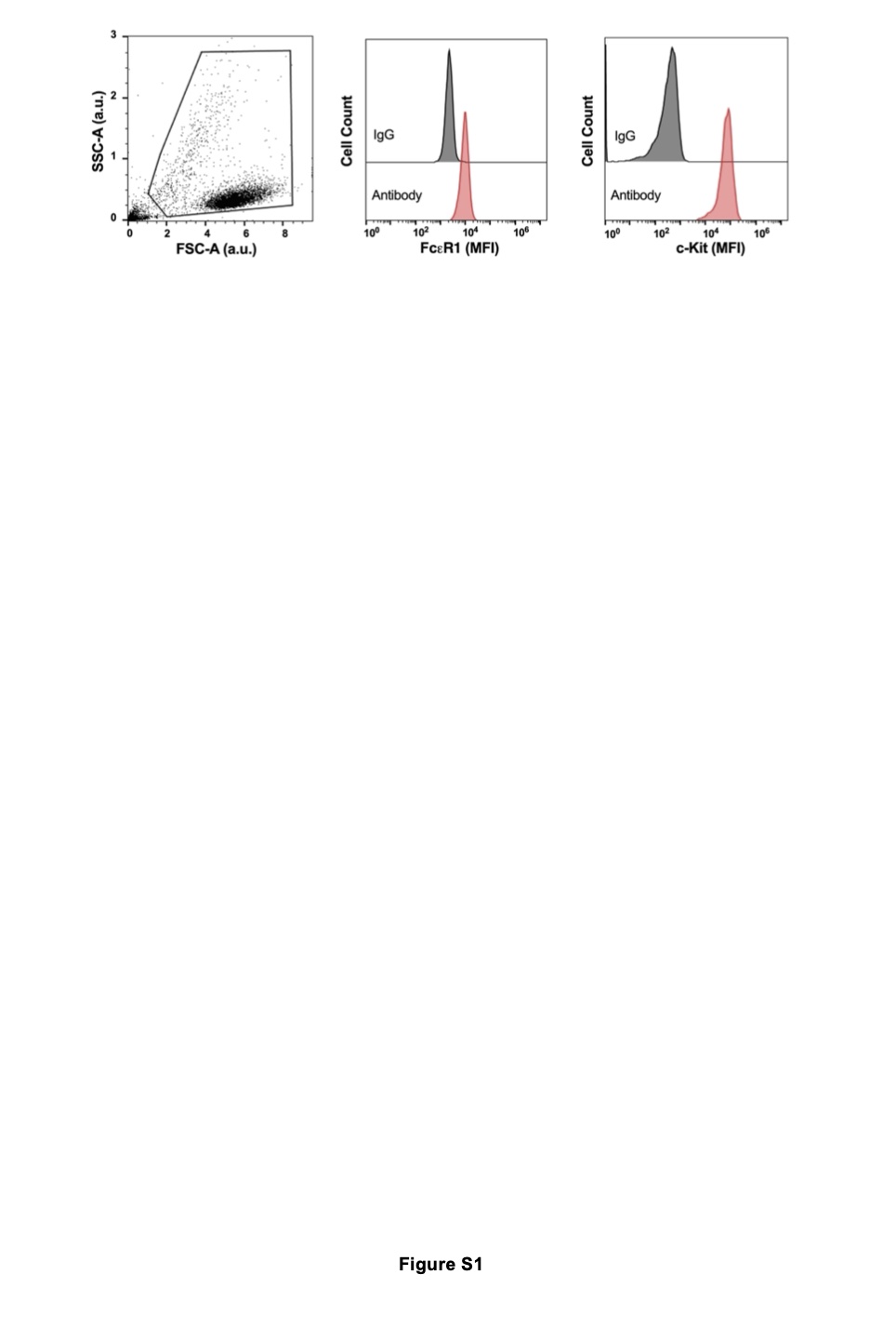

Supplement: Supplementary file 1 [file Image_1.jpeg]

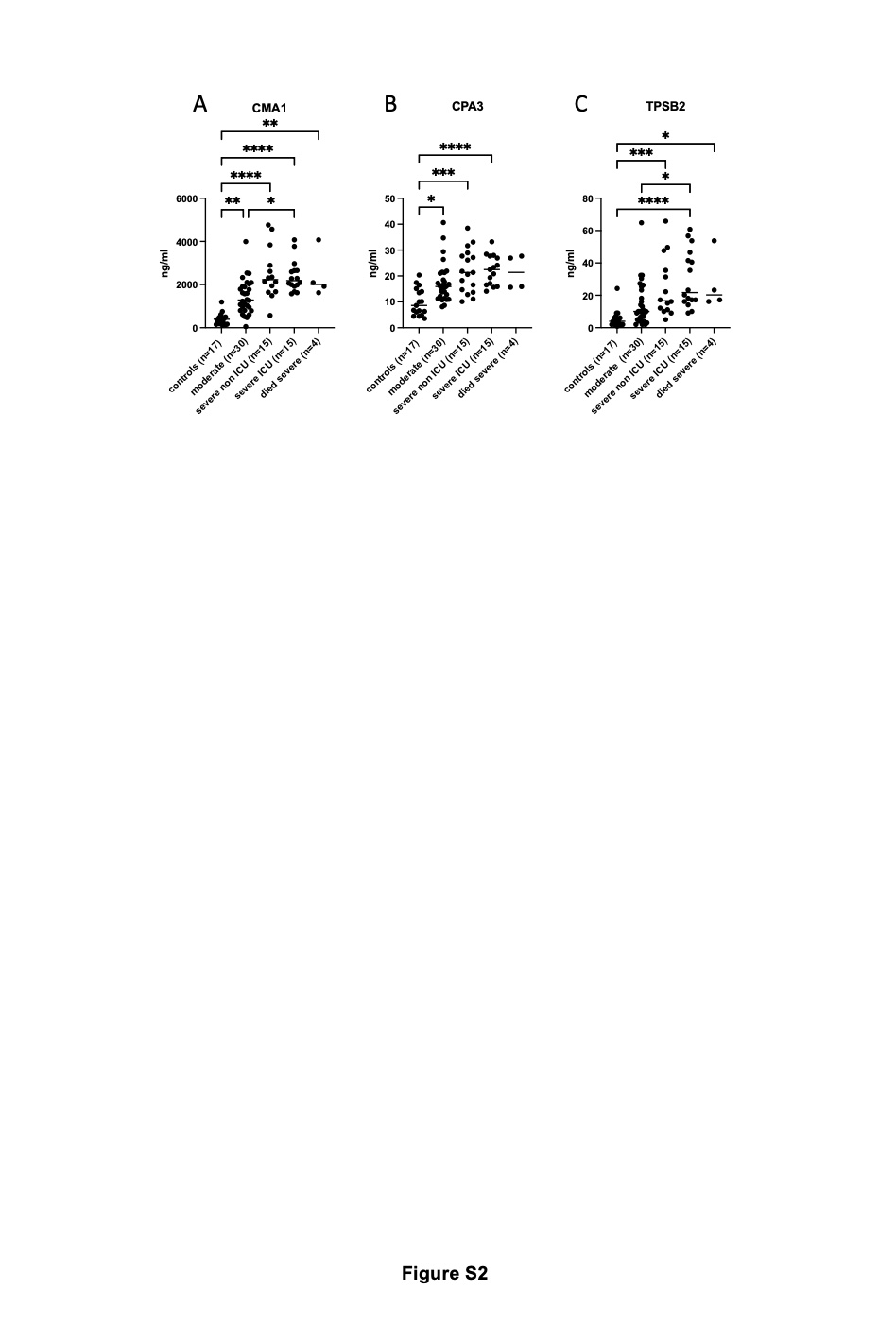

Supplement: Supplementary file 2 [file Image_2.jpeg]
